# Treatment with Light-Emitting Diodes of Wavelength 863 nm Delays DMBA/TPA-Induced Skin Tumor Formation and Decreases Proinflammatory Cytokine Levels in ICR Mice

**DOI:** 10.1155/2022/4400276

**Published:** 2022-02-23

**Authors:** Hyeong Ju Park, Yeon Hee Cho, Hyeyoon Goo, Sun-hyang Choi, Eun Young Kim, Namgue Hong, SangJoon Mo, Ji On Park, Byeong-Il Lee, Min Young Lee, Jin-Chul Ahn

**Affiliations:** ^1^Medical Laser Research Center, Dankook University, 31116, Republic of Korea; ^2^Department of Photobiology, Dankook University, Institute, 31116, Republic of Korea; ^3^Department of Medical Laser, Graduate School of Medicine, Dankook University, 31116, Republic of Korea; ^4^Pukyoung National University, 48513, Republic of Korea; ^5^Department of Otolaryngology-Head & Neck Surgery, College of Medicine, Dankook University, 31116, Republic of Korea

## Abstract

The popularity of light/energy devices for cosmetic purposes (e.g., skin care) is increasing. However, the effects and underlying mechanisms remain poorly understood. Commencing in the 1960s, various studies have evaluated the beneficial effects of a light source on cells and tissues. The techniques evaluated include low-level light (laser) therapy and photobiomodulation (PBM). Most studies on PBM used red light sources, but, recently, many studies have employed near-infrared light sources including those of wavelength 800 nm. Here, we used a light-emitting diode (LED) array with a wavelength of 863 nm to treat DMBA/TPA-induced mouse skin tumors; treatment with the array delayed tumor development and reduced the levels of systemic inflammatory cytokines. These results suggest that light therapy could be beneficial. However, the effects were small. Further studies on different skin tumors using an optimized LED setup are required. Combination therapies (conventional methods and an LED array) may be useful.

## 1. Introduction

In recent years, the interest in skin care has increased, given the desire for an ideal appearance, increase in disposable income, and people being more health-conscious. The skin is susceptible to light-induced, age-related deterioration [1]. To address this, dermatologists employ laser or light energy treatments. The popularity of light energy devices for cosmetic purposes (e.g., skin care) is increasing. However, the effects and underlying mechanisms remain poorly understood. Negative effects of lasers were reported in the late 1960s; since then, many studies have explored the positive effects of light sources on cells and tissues [2–4]. The techniques evaluated include low-level light (laser) therapy and photobiomodulation (PBM) employing light-emitting diodes (LEDs) [5]. Light photons are absorbed by photoreceptors such as chromophores and cytochrome C oxidase, promoting metabolism [3].

Light sources are commonly used to treat wounds and inflammation [6, 7]. Mo et al. used a light source of wavelength 630 nm to heal wounds and reported decreases in the levels of inflammatory cytokines (IL-1*β*, IL-6, and TNF-*α*) and growth factors (VEGF and TGF-*β*1) [7]. Hwang et al. used light sources of wavelengths 405, 532, and 650 nm to reduce the levels of inflammatory IL-8 secreted by human annulus fibrosus cells [8]. The applications are expanding to treatments for skin rejuvenation, lung inflammation, hair regrowth, and the repair of heart ischemic injury [5, 9]. Most PBM studies used red light sources; recently, many studies have explored near-infrared (NIR) light sources of 800 nm. Gupta et al. confirmed that laser light with a wavelength of 904 nm healed animal burns [10]. Höfling et al. treated thyroiditis patients with light of wavelength 830 nm—this improved thyroid function and reduced inflammation [11, 12].

Various pathologic skin lesions exist, and skin tumor is one of them. Surgical removal would be possible, but complications such as scar formation have to be considered when an invasive approach is employed. Thus, noninvasive therapeutic options are always welcomed and PBM would be one of the candidates. Here, we used an LED array of wavelength 863 nm to treat mouse skin tumors induced by 7,12-dimethylbenz(*α*)anthracene (DMBA) and tetradecaboylphorbol-13-acetate (TPA). This delayed tumor development and reduced the levels of systemic inflammatory cytokines.

## 2. Materials and Methods

### 2.1. Chemicals

**Figure 1:**
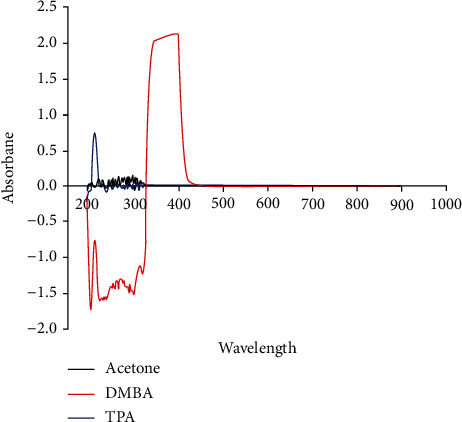
The results of the absorbance measurement with a spectrophotometer for acetone, DMBA, and TPA.

DMBA was used to initiate skin tumors, and TPA was employed to promote tumor growth; both were purchased from Sigma-Aldrich (USA), dissolved in acetone from Daejung Chemicals & Metals (Korea), and stored at 0°C. Then, absorbance was measured using a spectrophotometer (UV-1650PC, SHIMADZU) to confirm the reaction of chemicals by the light source. As shown in Figure 1, there was no absorption at the wavelength of 865 nm for each chemical (acetone, DMBA, and TPA).

### 2.2. Animals

Six-week-old female ICR mice (20–25 g) were purchased from Nara Biotech (Korea). All animal procedures adhered to the guidelines of the Institutional Animal Care and Use Committee of Dankook University (approval #DKU-20-009). The mice were allowed to acclimatize for 1 week with *ad libitum* access to pellet feed and water at 23 ± 1°C and a humidity of 44 ± 2% and under a 12 h light and 12 h dark cycle.

### 2.3. Experimental Design

**Figure 2:**
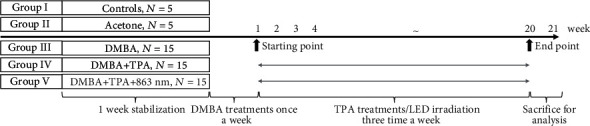
Experimental designs (group I: control, group II: acetone, group III: DMBA, group IV: DMBA+TPA, and group V: DMBA+TPA+863 nm LED).

A total of 55 ICR mice were randomly divided into five groups. The control (group I) and acetone (group II) groups included five animals each, and the DMBA (group III), DMBA+TPA (group IV), and DMBA+TPA+863 nm LED (group V) groups included 15 animals each. DMBA (60 *μ*g) and TPA (4 *μ*g) were dissolved in 200 and 500 *μ*L of acetone, respectively. DMBA was applied once (200 *μ*L) to the dorsa of mice in groups III, IV, and V. One week later, acetone and TPA were applied to the dorsum (500 *μ*L) three times weekly in groups II, IV, and V (Figure 2); mice in group V were LED-irradiated at the same time.

### 2.4. Irradiation

**Figure 3:**
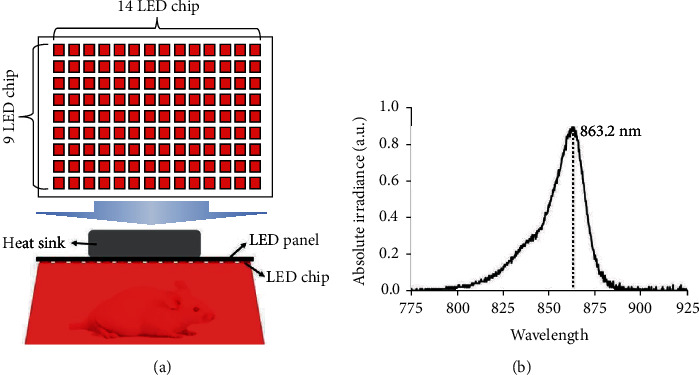
The LED panel and its absolute irradiance. (a) The panel featured 126 LED chips and was used to irradiate the mouse dorsum (8.5 mW/cm^2^, 30 min, 3 times weekly). (b) The light source central wavelength was 863.2 nm as measured by using a spectrometer.

The light source featured an 863 nm LED module (CL-SFC506IR-850, Ciel Light, Korea) and a 0.2 W LED chip (Table 1, Figure 3(a)). The LED spectrum was measured using a spectrometer (FLAME-T-VIS-NIR, Ocean Optics, USA; Figure 3(b)). The LEDs (*n* = 126; 14 × 9) were arranged on a printed circuit board of dimensions 14.6 × 21.0 cm. The 13.9 V power supply was a GPS-2303 device (GW Instek, Taiwan), and the power was measured using a photodiode power meter (PD300-TP-ROHS, Ophir Optronics, Israel). Mice in group V were irradiated for 30 min at 8.5 mW/cm^2^ immediately after TPA treatment.

### 2.5. Morphology

The mice were weighed, and their dorsa were imaged weekly. Tumor numbers were counted weekly. First, we calculated the percentages of mice with tumors as follows [13].

(1) $$\begin{array}{c} \frac{\text{Number of mice with tumor}}{\text{Total number of mic}}\times 100\%. \end{array}$$

Second, we calculated the average number of tumors per mouse. Tumor diameters were measured immediately before the mice were sacrificed at 20 weeks [14].

### 2.6. Histology Analysis

The dorsal skin was harvested from three random mice from each group and stored in 10% (*v*/*v*) formaldehyde. Skin samples were dehydrated through a graded series of ethanol baths, embedded in paraffin, and sectioned (4 *μ*m thick) using a microtome (RM2125, Leica, Germany). The sections were then stained with hematoxylin and eosin (H&E) and Picrosirius red (Junqueira et al. [1979]) [15, 16]. Epithelial thicknesses were measured, and areas of collagen were imaged using a microscope (BX53, Olympus, Japan) running cellSens software (Olympus). Collagenous areas were measured with the aid of ImageJ software (National Institutes of Health, USA) [17].

### 2.7. Immunohistochemistry (IHC) Analysis

The presence of cytokines in skin tissues adjacent to the tumor was visualized using IHC staining. The specimens were those used for histology; tissues embedded in paraffin were sliced into 4 *μ*m thick sections and mounted on slides that were incubated with polyclonal antibodies against rabbit anti-IL-6 (1 : 200, Abcam, UK) and rabbit anti-IL-1*β* (1 : 250, Abcam) overnight at 4°C. Then, the slides were incubated for 1 h at room temperature in a 1 : 1,000 dilution of secondary biotinylated anti-rabbit immunoglobulin G (Vector Laboratories, USA) and peroxidase-conjugated avidin (Vectastain Elite ABC Kit Standard PK-6105; Vector Laboratories) for 60 min [7], followed by incubation with DAB substrate and counterstaining with hematoxylin prior to microscopy (BX53, Olympus).

### 2.8. Statistical Analysis

All data are presented as the means ± standard deviations. All data underwent Shapiro-Wilk normality testing. Comparisons featured either one-way ANOVA or Kruskal-Wallis testing depending on data normality. Values of *P* < 0.05, *P* < 0.01, *P* < 0.001, and *P* < 0.0001 were considered to indicate statistical significance and are marked with the symbols ∗, #, or †. All data analyses employed Prism ver. 7 software (GraphPad Software Inc., USA).

## 3. Results

### 3.1. Morphological Observations

**Figure 4:**
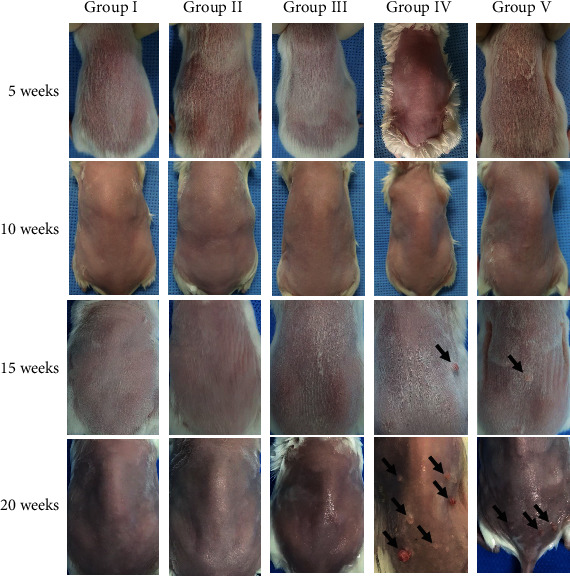
Representative images of mice in each group (group I: control, group II: acetone, group III: DMBA, group IV: DMBA+TPA, and group V: DMBA+TPA+863 nm LED) taken at weeks 5, 10, 15, and 20.

**Figure 5:**
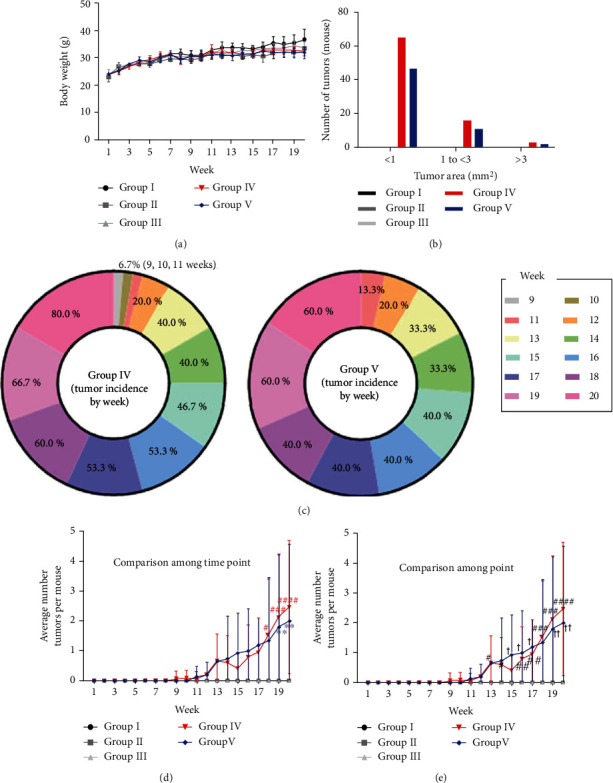
Effects of LED treatment on DMBA/TPA-induced mouse skin tumor formation (group I: control, group II: acetone, group III: DMBA, group IV: DMBA+TPA, and group V: DMBA+TPA+863 nm LED). (a) There was no among-group difference in mouse weight (error bar: standard deviation). (b) Tumors were classified by size (<1 mm^2^, 1 mm^2^ to <3 mm^2^, and >3 mm^2^). Group IV exhibited the widest size distribution. (c) Tumors first appeared at week 9 in group IV and week 11 in group V, and the numbers then gradually increased (to 80% in group IV and 60% in group V). (d, e) In the analysis of comparison among time points, significant differences compared to baseline were evident from week 18 in group IV and week 19 in group V. In the analysis of comparison among groups, post hoc significant differences compared to group I were evident from week 13 in group IV and week 15 in group V to the time of final measurements. ^####^*P* < 0.0001, ^###,^^∗∗∗^*P* < 0.001, ^##, ††^*P* < 0.01, and ^#, †^*P* < 0.05. Error bars: standard deviation.

Five groups were assessed in this study: the control (group I), acetone (group II), DMBA (group III), DMBA+TPA (group IV), and DMBA+TPA+863 nm LED (group V) groups. Tumors formed in mice in groups IV and V (Figure 4) but not in those in groups I, II, and III. Over the 20 weeks, the mouse weight showed a tendency to slightly decrease in groups IV and V compared to group I, but it was not statistically significant (Figure 5(a)). The first tumors (diameter < 1 mm) were observed at week 9 in group IV and week 11 in group V (Figure 5(b)). In both groups, the tumor size gradually increased and diversified from weeks 13 to 20. The tumors were graded by area as <1 mm^2^, 1 mm^2^ to <3 mm^2^, and>3 mm2. For all tumor sizes, the numbers for group IV were higher than those for group V (Figure 5(c)). The final tumor incidences (equation (1)) were 60% in group V and 80% in group IV (Figure 5(b)). The average numbers of tumors per mouse by time and group are shown in Figures 5(d) and 5(e). Significant difference of tumor incidence among time points first appeared at week 18 in group IV and week 19 in group V compared to the very beginning (Kruskal-Wallis test, *P* < 0.0001 (^####^*P* < 0.0001, ^###^*P* < 0.001, ^∗∗∗^*P* < 0.001, ^#^*P* < 0.05, and ^∗^*P* < 0.05); Figure 5(d), Table 2). In addition, in comparison among groups at each time point, the group I and IV data differed significantly from weeks 13 to 20 (Kruskal-Wallis test, *P* < 0.0001 (^####^*P* < 0.0001, ^###^*P* < 0.001, ^##^*P* < 0.01, and ^#^*P* < 0.05); Figure 5(e)). The group I and V data differed significantly from weeks 15 to 20 (Kruskal-Wallis test, *P* < 0.0001 (^††^*P* < 0.01, ^†^*P* < 0.05); Figure 5(e), Table 3). These results in overall suggest a statistically slower (difference occurs 19 weeks in group V, 19 weeks in group IV, at time point comparison; statistical group difference observed from 15 weeks in group V and 13 weeks in group IV) rate of skin tumor proliferation.

### 3.2. Histological Assessment

**Figure 6:**
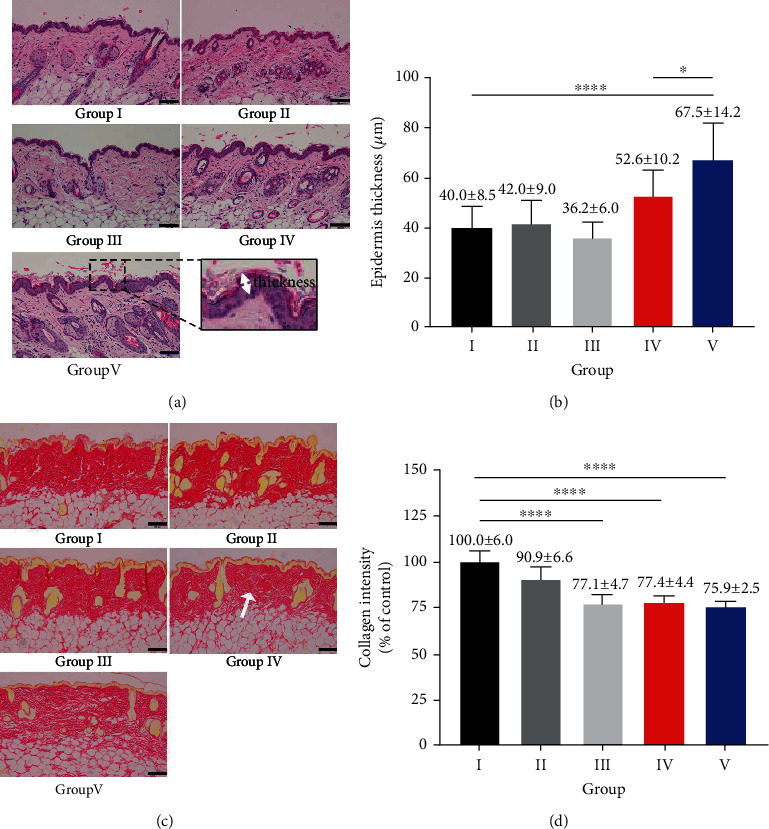
Histological results (group I: control, group II: acetone, group III: DMBA, group IV: DMBA+TPA, and group V: DMBA+TPA+863 nm LED). (a) Images of the H&E-stained skin around tumors in each group (scale bars (black): 100 *μ*m) are shown. The epidermal thicknesses (as depicted by arrow) were measured in each group. (b) Average epidermal thicknesses of each group are shown. The values for groups IV and V were 52.6 ± 10.2 and 67.5 ± 14.2 *μ*m, respectively. Epidermal thickness was statistically thicker in group V compared to groups I and IV. (c) Images show the collagen densities (white arrows) of the skin around tumors as determined using Picrosirius red staining (scale bars (black): 100 *μ*m). (d) The collagen staining intensity was significantly lower in groups IV and V compared to group I. ^∗∗∗∗^*P* < 0.0001, ^∗^*P* < 0.05. Error bars: standard deviations.

Epidermal thicknesses near the tumors were measured via H&E staining.  Figure 6(a) shows a representative image; the thickness of the region indicated by the yellow circle in the group IV image (Figure 6(a)) was measured. The mean thicknesses were 40.0 ± 8.5 *μ*m (group I), 42.0 ± 9.0 *μ*m (group II), 36.2 ± 6.0 *μ*m (group III), 52.6 ± 10.2 *μ*m (group IV), and 67.5 ± 14.2 *μ*m (group V) (Figure 6(b)). Thus, the epidermis was thicker in groups IV and V, which received the TPA and TPA+850 nm LED treatments, respectively, than in groups I, II, and III. In particular, the epidermal thickness in group V was 1.7-fold that of group I and 1.2-fold that of group IV (one-way ANOVA, *F* = 12.86, *R*‐squared = 0.595, *P* < 0.0001; Bonferroni's multiple comparison test; group I vs. group V (*P* < 0.0001), group IV vs. group V (*P* < 0.05); Figure 6(b)).

Picrosirius red staining was performed to measure changes in dermal collagen density (Figures 6(c) and 6(d)). The densities in the regions indicated by the white arrow in the representative image from group IV were measured, with the density of group I taken to be 100%. In group V, the density was 75.9 ± 2.5% (Figure 6(d)). The decrease of 24.1% compared to group I was significant (one-way ANOVA, *F* = 22.58, *R*‐squared = 0.8187, *P* < 0.0001; Bonferroni's multiple comparison test; group I vs. group III (*P* < 0.0001), group I vs. group IV (*P* < 0.0001), and group I vs. group V (*P* < 0.0001); Figure 6(d)).

### 3.3. IHC Analysis

**Figure 7:**
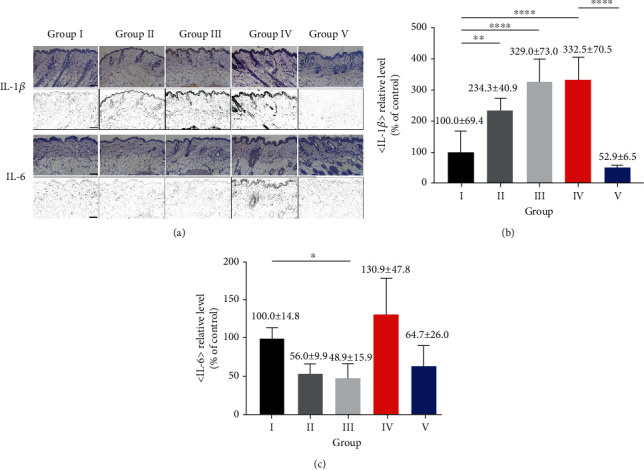
IHC analysis of inflammatory cytokine (IL-1*β*, IL-6) (group I: control, group II: acetone, group III: DMBA, group IV: DMBA+TPA, and group V: DMBA+TPA+863 nm LED). (a) Images of IL-1*β* and IL-6 expressions among the adjacent skin close to the tumor are shown (first and third rows). And the images in the second and fourth rows show the results extracted using FIJI ImageJ (provided by the National Institutes of Health) software to analyze the expression levels of IL-1*β* and IL-6. (b) DAB intensities were extracted and quantified. The IL-1*β* level was higher in groups II, III, and IV compared to the other groups but lower in group V compared to groups I, II, III, and IV. (c) The IL-6 level was higher in group IV compared to group I but somewhat lower in group V compared to groups I and IV; however, statistical significance was lacking. ^∗∗∗∗^*P* < 0.0001, ^∗∗^*P* < 0.01, and ^∗^*P* < 0.05. Error bar: standard deviation.

IHC analysis was used to measure the levels of inflammatory cytokines in tissues. The DAB staining intensities were measured (Figure 7(a)). The IL-1*β* levels were 100.0 ± 69.4% in group I, 234.3 ± 40.9% in group II, 329.0 ± 73.0% in group III, 332.5 ± 70.5% in group IV, and 52.9 ± 6.5% in group V (Figure 7(b)). The IL-1*β* level was higher by 232.5% in group IV compared to group I, whereas, in group V, it was lower by 47.1% compared to group I. Thus, significant differences were evident between groups I and IV and groups IV and V (one-way ANOVA, *F* = 29.64, *R*‐squared = 0.8259, *P* < 0.0001; Bonferroni's multiple comparison test; group I vs. group II (*P* < 0.01), group I vs. group III (*P* < 0.0001), group I vs. group IV (*P* < 0.0001), and group IV vs. group V (*P* < 0.0001); Figure 7(b)).

The IL-6 levels were 100.0 ± 14.8% in group I, 56.0 ± 9.9% in group II, 48.9 ± 15.9% in group III, 130.9 ± 47.8% in group IV, and 64.7 ± 26.0% in group V (Figure 7(c)). The group IV level was the highest. The group V level was slightly higher than those of groups II and III but 35.3% lower than that of group I and 66.2% lower than that of group IV. There was difference among groups. Post hoc analysis showed statistical differences between group I and groups II and III, not between group I and groups IV and V (Kruskal‐Wallis statistic = 20.03, *P* = 0.0005; Dunn's multiple comparison test; group I vs. group III (*P* < 0.05); Figure 7(c)).

## 4. Discussion

PBM facilitates angiogenesis, hair follicle regeneration, collagen production, and the reduction of inflammation and edema [1, 2, 4]. Red and NIR wavelengths exhibit excellent transmittance [6, 7, 18, 19]. However, light treatments have been rarely used in patients with neoplasm [20]. Rhee et al. injected FRO thyroid cancer cells into the mouse thyroid followed by irradiation with a 650 nm diode laser. But anaplastic thyroid carcinoma overproliferation and angiogenesis were observed [21]. Moteiro et al. found that DMBA-induced tumors in the hamster cheek pouch grew after irradiation with a 660 nm laser [22]. Few studies have investigated the effects of light sources of IR and NIR wavelengths on tumor formation and expansion. Previous studies used light energy wavelength between 650 and 660 nm, and they reported aggravated tumor growth which is opposite to the outcome of the current study showing delay of tumor growth and decreased inflammatory cytokine.

We used a two-stage model employing the DMBA carcinogen and TPA promoter [23] to rapidly establish stable tumors in mice [24]. Tsai et al. reported epidermal thickening after TPA application [25]. Similarly, in our study, the epidermal thicknesses in TPA-treated groups were greater than those in group I (control). But unexpectedly, histological examination revealed that the thickest epidermis was obtained after irradiation (863 nm LEDs; Figures 6(a) and 6(b)). However, tumor formation was slower in group V than in group IV. This result does not match with the theory that tumor proliferation would correlate with the epidermal thickness [25, 26]. Since the epidermis is the part that receives light energy by PBM directly, increased proliferation of epidermal tissue might have led to increased skin thickness. This can be explained by the results reporting that near-infrared light can regulate the epithelial proliferation rate and the skin immune system [27, 28]. Nevertheless, further studies using this specific light wavelength and histology of the skin epidermis are necessary.

We also measured collagen densities. Increased collagen degradation and decreased biosynthesis perturb collagen homeostasis [29–31] in human degenerative diseases and cancers, with collagen lost overall [32]. Local collagen degradation accompanies tumor formation and metastasis [33, 34]; also, matrix metalloproteinase in aging skin degrades collagen [31]. Picrosirius red staining confirmed that the collagen density in groups IV and V differed significantly from that in group I (Figure 6(d)). However, since it is not clear whether these neoplastic lesions are malignant, we might speculate the possibility of malignant change in both groups (IV and V). The collagen density did not improve upon NIR irradiation (850 nm). But considering the relatively small improvement by PBM in tumor growth, this insufficient outcome is understandable.

Inflammation occurs as tumors develop and proinflammatory cytokines (COX-2, iNOS, IL-1*β*, IL-6, interferon-*γ*, TGF-*β*, and IL-8) are activated [35–37]. IL-1*β* is secreted at the time of tumor initiation, and the level then decreases, but that of IL-6 rises [38]. IL-6 at low levels serves as a defense; at higher levels, it induces inflammation [37]. In group IV, both the IL-1*β* and IL-6 levels were high (Figure 7). Group V exhibited lower levels, suggesting that the NIR light treatment reduced inflammation [39, 40]. Furthermore, this reduced inflammation which is supported by many prior publications using PBM could be the possible mechanism for delayed tumor proliferation by NIR light energy. Similarly, in this study, the level of cytokines decreased in the group irradiated with a light source with a wavelength of 865 nm. Walski et al. reported that the R/NIR irradiation inhibited neutrophil infiltration and decreased cytokines in the animal in vivo models [39]. It has proven to have anti-inflammatory effects. However, the tumor remains and cytokines are present in the surrounding tissues. Therefore, it is considered that there is potential for rebound tumor growth by the remaining tumor. Finally, continuous irradiation with NIR light sources requires further long-term studies of tumor suppression/growths.

## 5. Conclusions

In summary, a light source of wavelength of 863 nm slowed tumor development and the associated inflammation. These results suggest that light/energy therapy may be useful. However, the beneficial effects were small. Further studies using different skin tumors and optimized LEDs are required. In particular, combination therapies using conventional methods and LED illumination may be valuable.

## Figures and Tables

**Table 1:** Specifications of the irradiation light source.

Item	LED
Center wavelength	863.6 nm
Bandwidth	100 nm
Full width at half maximum (FWHM)	30 nm
Operating mode	Continuous wave (CW)

**Table 2:** Statistical analysis of comparison among time points in the average number of tumors per mouse (group I: control; group II: acetone; group III: DMBA; group IV: DMBA+TPA; group V: DMBA+TPA+850 nm LED; KW^1)^: Kruskal-Wallis statistic).

Week	Group IV			Group V		
	*P* value	KW^1)^	Dunn's multiple comparison test	*P* value	KW^1)^	Dunn's multiple comparison test
1 vs. 2	<0.0001	126	ns	<0.0001	90.9	ns
1 vs. 3			ns			ns
1 vs. 4			ns			ns
1 vs. 5			ns			ns
1 vs. 6			ns			ns
1 vs. 7			ns			ns
1 vs. 8			ns			ns
1 vs. 9			ns			ns
1 vs. 10			ns			ns
1 vs. 11			ns			ns
1 vs. 12			ns			ns
1 vs. 13			ns			ns
1 vs. 14			ns			ns
1 vs. 15			ns			ns
1 vs. 16			ns			ns
1 vs. 17			ns			ns
1 vs. 18			#			ns
1 vs. 19			###			∗∗
1 vs. 20			####			∗∗

**Table 3:** Statistical analysis of comparison among groups in the average number of tumors per mouse (group I: control; group II: acetone; group III: DMBA; group IV: DMBA+TPA; group V: DMBA+TPA+850 nm LED; KW^1)^: Kruskal-Wallis statistic) (there was no statistical significance from 1 week to 12 weeks).

Week	*P* value	KW^1)^	Dunn's multiple comparison test	Dunn's multiple comparison test
			Group I vs. group IV	Group I vs. group V
13	0.0032	15.86	#	ns
14	0.0007	19.16	#	ns
15	0.0003	21.13	ns	†
16	<0.0001	25.52	##	†
17	<0.0001	25.56	##	†
18	<0.0001	28.75	###	ns
19	<0.0001	36.96	###	††
20	<0.0001	42.94	####	††

## Data Availability

All relevant data are contained within the article.
